# Holding the line: predictors of coronal and sagittal loss of reduction after tibial plateau fracture fixation

**DOI:** 10.1007/s00402-026-06437-7

**Published:** 2026-07-28

**Authors:** Mees K. Hesmerg, Joyce L. Benner, Pieter Joosse, Lucien C.M. Keijser

**Affiliations:** 1 Amsterdam Movement Sciences, Department of Orthopedic Surgery and Sports Medicine, Amsterdam University Medical Centers, Amsterdam, Netherlands; 2 Department of Orthopaedic Surgery, Noordwest Ziekenhuisgroep, Alkmaar, The Netherlands; 3 Department of Trauma Surgery, Noordwest Ziekenhuisgroep, Alkmaar, The Netherlands

**Keywords:** Tibial plateau fracture, Loss of reduction, Secondary displacement, Risk factors, Regression analysis

## Abstract

**Introduction:**

Tibial plateau fractures (TPFs) are challenging fractures that often suffer from secondary Loss of Reduction (LoR) after fracture fixation. In this study, we aimed to investigate the factors and characteristics associated with LoR after surgical fixation, using measurements of both coronal and sagittal displacement.

**Methods:**

A retrospective analysis of 213 TPF patients (79.3% female, mean age 57.5 ± 14.4 years) surgically treated between 2017 and 2024 in a level 1 trauma center was performed. Patients were included if they had a minimum of 100-days radiological follow-up. Loss of reduction was defined as late postoperative depression ≥ 3 mm, condylar widening ≥ 5 mm, varus/valgus or tibial slope collapse > 5 degrees, breakage, loosening, or conversion to total knee arthroplasty (TKA). Radiographic images were assessed for preoperative, direct postoperative, and late postoperative (up to 1 year) displacement. Univariable and multivariable logistic regression analyses were employed to study factors associated with LoR..

**Results:**

At a mean follow-up of 325 days (SD ± 161) 77 patients (36.2%) suffered from LoR. The adjusted multivariable logistic regression model revealed that patient age (OR 1.03, *p* = 0.028), involvement of the posterior column (OR 2.87, *p* = 0.014), preoperative step-off (OR 1.08, *p* < 0.001), postoperative step-off (OR 1.61, *p* = 0.007), and postoperative widening (OR 1.31, *p* = 0.003) were independently associated with higher odds of LoR.

**Conclusion:**

Using strict radiological criteria, patient age, posterior column involvement, greater preoperative step-off, and residual step-off and condylar widening were associated with increased odds LoR. These findings underscore the importance of achieving optimal fracture reduction and fixation to minimize the risk of LoR, especially in older patients and complex posterior fracture patterns.

## Introduction

 Tibial plateau fractures are complex, intra-articular knee injuries that often require surgical fixation. They occur most frequently in young males following high-energy trauma mechanisms and in older females after low-energy trauma mechanisms. The incidence is estimated at approximately 10–15 per 100,000 annually [1–3]. Management typically consists of open reduction and internal fixation (ORIF) or conservative treatment, such as bracing or casting [4]. Despite advances in fixation techniques, these fractures are widely regarded as technically demanding, and loss of reduction (LoR) remains a major concern. Reported LoR rates range from approximately 10% to 30% in surgically treated patients [5–8]. Postoperative LoR is associated with reduced range of motion, pain, and long-term posttraumatic osteoarthritis [9].

Previous studies have explored risk factors associated with LoR, including fracture morphology, patient characteristics, and surgical techniques [6, 7, 10]. However, their findings are contradictory. Two studies highlight the relevance of fractures in the posterior coronal plane and employ a limited set of discrete patient characteristics [6, 10], another disputes this and instead emphasizes the use of preoperative displacement measurements to assess the risk of LoR [7]. All studies to date have assessed LoR only in the coronal plane on anterior-to-posterior oriented radiography, using tibial plateau angulation relative to the tibial axis. No study has yet evaluated the influence of preoperative displacement on the risk of LoR in both the coronal and sagittal planes, incorporating radiographic characteristics such as plateau widening, slope, or step-off, alongside patient-related factors.

The aim of this study was to identify patient-, fracture- and reduction-related factors associated with LoR, using strict radiological criteria beyond varus/valgus alignment in a large cohort of surgically treated patients in a level I trauma center.

## Methods

This retrospective cohort study was conducted in a level I trauma center in the Netherlands and included patients who were presented and surgically treated between January 2017 and October 2024. Prior to commencement, institutional review board approval was obtained.

### Study population

Potentially eligible patients were identified through searches in the electronic health records (EHR) using CTcue (CTcue B.V., Amsterdam, the Netherlands), a self-service data mining tool employing artificial intelligence, enabling the searching, extraction and structuring of EHR data. The search included a combination of diagnostic codes and corresponding surgical procedure codes. Results gathered from CTcue are subsequently cross-referenced using EHR to validate their eligibility and output. Patients were eligible for inclusion if they (1) were surgically treated for a tibial plateau fracture (OTA/AO 41B or OTA/AO 41 C), (2) had at least 100 days of follow-up, including radiographic imaging, and (3) were above the age of 18 at the time of injury. Patients were excluded if they (1) suffered from direct postoperative residual malalignment that surpassed the predefined threshold for radiologic failure as given by Ali et al. [8], i.e. direct postoperative step-off ≥ 3 millimeters or condylar widening ≥ 5 millimeters, (2) had active malignant disease at the time of fracture, (3) had a history of rheumatoid arthritis, (4) were unable to comply with postoperative weight bearing restrictions, or (5) had objected to medical research in the past.

### Patient and radiographic characteristics

Patient characteristics were retrieved from the EHR database and included: age at fracture, sex, BMI, comorbidity (renal disease, pulmonary disease, cardiovascular disease, use of systemic corticosteroids, presence of diabetes mellitus), active smoking status at time of fracture, T-score on dual-energy X-ray absorptiometry (DEXA) scan. According to national guidelines, DEXA scans are offered on a voluntary basis to patients aged 50 years or older as part of routine care. Surgical characteristics included fixation method (screw-only, single plating, dual plating), use of subchondral rafting constructs, and bone grafting. To assess whether potential temporal changes in surgical technique during the study period were associated with a change in LoR, year of surgery was included as a variable.

Preoperative images, direct postoperative images, and late images (100 days to 1 year follow-up) were measured using standardized methods previously described by Assink et al. [11]. Plain radiographs and Computed Tomography (CT) scans were used to assess fracture configuration and displacement. CT imaging is routinely collected for all patients who undergo surgical fixation of TPFs and was therefore available for all included patients. The following measurements were taken: step-off, condylar widening, medial proximal tibial angle (MPTA), and tibial slope. The fracture configuration was assessed on CT imaging using the OTA/AO classification [12] and the revised Three-column classification by Hoekstra et al. [13], which divides the transversal tibial plateau into four distinct sections or columns: medial, lateral, posterior, and posterolateral. Furthermore, comminution was assessed, defined as more than three major fragments [7], and the presence of a fracture in the coronal plane was assessed, in which the major fracture line of the medial or lateral articular surface was within − 45° to 30° of the posterior femoral condylar axis [6, 7, 10].

### Secondary loss of reduction

The primary outcome was defined as reaching a predefined, clinically relevant threshold of radiographic displacement on late postoperative imaging, rather than the absolute magnitude of secondary displacement from the immediate postoperative radiograph. Secondary LoR resulting in failure of fixation was defined according to the proposed thresholds of Ali et al. [8]. Failure included a condylar widening of 5 millimeters or greater, depression of 3 millimeters or greater, varus- or valgus collapse of more than 5 degrees, or tibial slope collapse of more than 5 degrees. Other modes of failure included breakage, loosening, or conversion to total knee arthroplasty (TKA). All measurements were performed by a single rater (MH, orthopedic surgery resident) using these standardized definitions. To assess inter-rater reliability, a second independent rater (LK, consultant orthopedic surgeon) repeated the measurements in a subset of consecutive patients (*n* = 25). The second rater was blinded to the measurements of the primary rater.

#### Statistical analysis

Patient characteristics were studied using descriptive statistics. Inter-rater reliability was assessed using intraclass correlation coefficients (ICCs) with 95% confidence intervals (95% CI) using a two-way random effects model for absolute agreement. To identify factors associated with LoR, logistic regression analysis was used. First, a univariable prescreening approach was applied to select variables to be included in the multivariable regression model. Variables were included in the multivariable model if the p-value was < 0.25 [14]. Next, a multivariable logistic regression analysis was run using all previously identified variables of interest using a backward selection likelihood ratio (LR) procedure. Lastly, the final multivariable model was checked for confounding using a forced entry approach; variables were considered confounders if they yielded a beta-coefficient change of 10% or greater in any of the variables in the multivariable model. All analyses were conducted using IBM SPSS statistics (Version 28). No imputation of missing data was performed. Analyses of variables with missing data were performed as available-case analyses. Accordingly, the univariable analysis of DEXA-derived T-scores was restricted to a limited subset of patients (*n* = 84). For the final model, an alpha value of *p* < 0.05 was used.

## Results

**Fig. 1:**
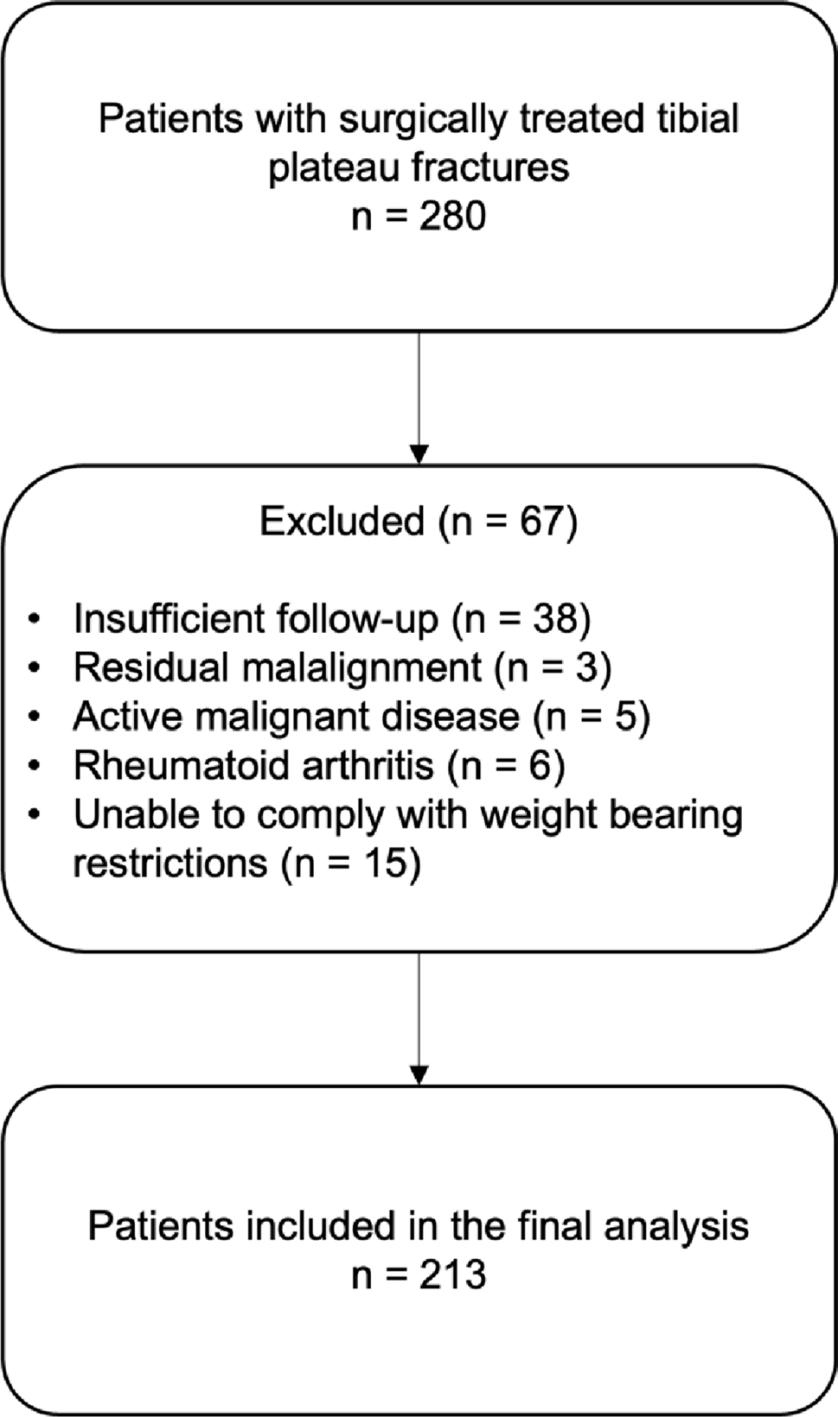
Flow diagram of patient selection procedure

Between 2017 and 2024, a total of 280 patients were surgically treated for a TPF. After initial assessment, 67 were excluded from the study. For 38 patients, there was insufficient follow-up available. Furthermore, there were 3 cases of residual malalignment, 5 cases of active malignant disease, 6 patients with rheumatoid arthritis, and 15 patients could not comply with postoperative weight-bearing restrictions due to comorbidity or concomitant injury. This resulted in a total of 213 patients who were included in the analysis (Fig. 1). Baseline patient characteristics are given in Table 1. In total, there were 77 patients who suffered from LoR (36.2%).

**Table 1.** Patient characteristics (n=213)

Sex, female	169 (79.3%)
Age (years)*	57.5 ± 14.4
Follow-up (days)*	325 ± 161
BMI (kg/m^2^)*	26.7 ± 5.0
Renal disease	7 (3.3%)
Pulmonary disease	9 (4.2%)
Cardiovascular disease	59 (27.7%)
Systemic corticosteroid use	5 (2.3%)
Type I Diabetes mellitus	2 (0.9%)
Type II Diabetes mellitus	19 (8.9%)
Active smoker	32 (15.0%)
T-score (*n* = 84)*	−2.4 ± 0.83
Comminuted fracture	95 (44.6%)
Coronal fracture	63 (29.6%)
AO/OTA Classification	
41B1	12 (5.6%)
41B2	11 (5.2%)
41B3	105 (49.3%)
41C1	21 (9.9%)
41C2	9 (4.2%)
41C3	55 (25.8%)
Posterior column involvement	101 (47.4%)
Lateral column involvement	189 (88.7%)
Medial column involvement	81 (38.0%)
Posterolateral column involvement	128 (60.1%)
Fixation method	
Screw-only fixation	37 (17.4%)
Single plating	112 (52.6%)
Dual plating	64 (30.0%)
Use of rafting construct	44 (20.7%)
Use of bone graft	54 (25.4%)

* Given as mean ± SD

The inter-rater reliability of the radiographic measurements was assessed using 25 consecutive cases and showed good ICCs for all measurements: step-off (ICC = 0.758, 95% CI 0.450–0.894), condylar widening (ICC = 0.890, 95% CI 0.754–0.951), MPTA (ICC = 0.830, 95% CI 0.594–0.927) and slope (ICC = 0.791, 95% CI 0.534–0.907).

The univariable prescreening analysis identified several variables of interest, all variables with a p-value < 0.25 are presented in Table 2. Sex (*p* = 0.503), BMI (*p* = 0.343), renal disease (*p* = 0.673), pulmonary disease (*p* = 0.598), presence of diabetes mellitus (*p* = 0.336), systemic steroid use (*p* = 0.856), active smoking (*p* = 0.821), T-score on DEXA imaging (*p* = 0.971), and use of rafting constructs (*p* = 0.974) did not meet the predefined screening threshold and were not included in the multivariable analysis. Changes over time were explored using the year of surgery as a continuous variable in a univariable logistic regression as well, no significant association was found (*p* = 0.474). The final multivariable model found that age, posterior column involvement, preoperative step-off, postoperative step-off, and postoperative condylar widening had a significant relationship with LoR. Comminution was found to be the only significant confounder and was included in the adjusted model (Table 2).

**Table 2:** Univariable, multivariable, and adjusted multivariable regression analyses of factors associated with loss of reduction

Variable	Univariable analysis			Unadjusted multivariable analysis			Adjusted multivariable analysis*		
	OR	95% CI	*p*-value	OR	95% CI	*p*-value	OR	95% CI	*p*-value
Age, years	1.02	0.99–1.04	0.060	1.03	1.00–1.06	0.024	1.03	1.00–1.06	0.028
Cardiovascular comorbidity	1.44	0.78–2.67	0.243						
Fixation method									
Single plating	Ref	Ref	Ref						
Screw fixation	0.61	0.25–1.46	0.265						
Dual plating	2.54	1.36–4.70	0.005						
Use of bone graft	1.97	1.05–3.69	0.035						
Coronal fracture	1.99	1.09–3.64	0.025						
Comminution	3.42	1.91–6.14	< 0.001						
Posterior column involved	4.05	2.23–7.35	< 0.001	3.46	1.68–7.13	< 0.001	2.87	1.24–6.65	0.014
Total number of columns	1.68	1.31–2.16	< 0.001						
Preoperative step-off, mm	1.11	1.07–1.16	< 0.001	1.09	1.04–1.14	< 0.001	1.08	1.03–1.14	< 0.001
Preoperative widening, mm	1.16	1.09–1.23	< 0.001						
Preoperative MPTA, degrees	1.13	1.04–1.22	0.003						
Preoperative slope, degrees	1.05	1.00–1.10	0.043						
Postoperative step-off, mm	1.90	1.41–2.55	< 0.001	1.66	1.17–2.35	0.004	1.61	1.24–2.28	0.007
Postoperative widening, mm	1.42	1.22–1.66	< 0.001	1.30	1.09–1.56	0.004	1.31	1.09–1.57	0.003
Postoperative MPTA, degrees	1.24	1.07–1.43	0.005						

Continuous variables are given as *‘Variable*,* unit of increase’*

*OR* Odds ratio, *CI* Confidence Interval

*The adjusted multivariable analysis was adjusted for comminution

## Discussion

The aim of this study was to assess which patient-, fracture-, and reduction- related characteristics are associated with LoR of surgically treated tibial plateau fractures. LoR was found in 36.2% of the patients. The multivariable logistic regression analysis found that age, posterior column involvement, preoperative step-off, postoperative step-off, and postoperative condylar widening showed an independent relationship with LoR after adjustment for comminution.

In this study, age was the only patient-related characteristic that showed a significant relationship with LoR. Although age was previously suspected to be associated with LoR [8], previous regression analyses have not reached statistical significance [6, 7]. However, a strong trend towards an association was observed in the study by Shimizu et al., with very similar results (OR 1.04, 95% CI: 1.00–1.07, *p* = 0.051) to the results in this study (OR 1.03, 95% CI: 1.00–1.06, *p* = 0.028). As the age of a human progresses, fracture healing is impaired. Osteoblast activity is decreased, mesenchymal cell activity is diminished, and the bone matrix undergoes several alterations [15, 16]. Angiogenesis is decreased, resulting in decreased oxygen and nutrition supply at the fracture site [17, 18]. This may lead to smaller and weaker callus formation, which could subsequently lead to higher rates of LoR.

Posterior fractures of the tibial plateau are a well-known surgical challenge due to limited access to, and visualization of posterior fragments [19]. This study assessed involvement of the posterior column as an independent variable, separate from coronal fracture patterns. The study by Kim et al. [6] reported that a posterior approach should be considered if a posterior coronal fracture is present, but did not report separately on the involvement of the posterior column. In this study, it was found that not the coronal fracture, but the involvement of the posterior column itself was associated with LoR. This suggests that increased attention is necessary in all fractures involving the posterior column, not only those involving a major coronal fracture line. From a surgical perspective, this emphasizes the importance of adequate reduction and stabilization of posterior fragments. Depending on fracture morphology, posterior approaches may have to be considered to provide adequate support. This study was not designed to compare surgical approaches, and thus no conclusions regarding superiority of a particular strategy can be drawn.

The amount of preoperative step-off has previously been described as a factor associated with LoR, as it may reflect the severity of a fracture and collapse or comminution of the subchondral cancellous bone, resulting in loss of mechanical support for the articular surface [7, 8, 20]. Even after fixation, the weakened subchondral structures may fail to withstand load-bearing, and LoR may occur. The mechanical consequences of loss of subchondral support could also be influenced by the employed strategy for fixation. Additional techniques such as subchondral rafting constructs, bone grafting, or more robust constructs were statistically explored but showed no independent association with LoR in the multivariable model. It has also been associated with concomitant soft tissue injury, possibly changing the kinematic axis of the knee joint and increasing stress on the involved plateau [21, 22]. An articular depression of > 6 mm is also independently associated with conversion to TKA [11], further confirming that it plays an important role in LoR.

The finding that residual postoperative coronal displacement in step-off or widening is associated with increased LoR highlights the distinction between direct postoperative malreduction and subtle residual displacement. Patients with immediate malreduction were excluded from the study. Nevertheless, subtle residual displacement below the predefined threshold remained associated with an increased risk of subsequently reaching LoR. This suggests that not only gross malreduction but also subtle residual displacement may increase the risk of LoR.

This association could be explained by altered biomechanical loading of the knee. Residual step-off can cause a concentration of contact stress and abnormal shear forces, leading to uneven load distribution and increased contact pressure on the plateau [23]. Increased postoperative condylar widening changes the geometry of the knee joint and may alter the weight distribution on the tibial plateau. It also may cause the femur to suffer from increased coronal instability due to its ability to shift laterally or medially on the tibial plateau, causing laxity and increasing weight on inadequately buttressed fracture portions. The study by Shimizu et al. [7] found similarly that postoperative widening was a risk factor for loss of alignment of the tibial plateau, relative to the tibial axis. They found no significant relationship with residual articular step-off. This finding underscores the importance of achieving accurate anatomical fracture reduction to maintain postoperative alignment.

This study has used strict radiological criteria to assess both coronal and sagittal displacement using measurements in step-off, widening, MPTA, and tibial slope. The relatively high incidence (36.2%) may be explained, in part, by the radiologic criteria for LoR. Whereas previous studies have focused on coronal alignment or a limited number of radiological criteria, this study assessed both coronal and sagittal displacement and multiple manifestations of fixation failure, including LoR, implant failure, and conversion to TKA. Consequently, our definition may capture subtle LoR that may have not been classified as LoR in earlier studies. These criteria are based on the study by Ali et al. [8], and the clinical relevance of the cutoff points has been confirmed in previous studies. For instance, a step-off of more than 2 millimeters is associated with the development of posttraumatic osteoarthritis [24], and patients with a step-off below 2.5 millimeters have a greater range of motion and score better on several patient-reported outcome measures [25]. Coronal and sagittal malalignment of more than 5 degrees, measured through MPTA and tibial slope, is associated with a higher risk of posttraumatic osteoarthritis and risk of conversion to TKA [11, 24]. Lastly, plateau widening of 5 millimeters or more was also associated with increased risk of conversion to TKA, reflecting a negative clinical outcome [11]. By using these criteria, this study has aimed to assess LoR using a comprehensive and reliable method with objective measurements that also have clinical relevance.

### Strengths and limitations

This study has several strengths. It considers the sagittal plane in LoR, and it has a relatively large sample size, with over 200 patients included in the analysis. The objective measurements allow for repeatability and standardized outcome measurement. By using a minimum follow-up of 100 days, it was aimed to ensure sufficient postoperative surveillance to detect early LoR, whilst allowing the fracture to go through the initial phase of articular healing. There are also some limitations to consider in this study. First and foremost, the retrospective nature should be addressed. It is possible that patients are lost to follow-up before the secondary displacement occurs. It is unknown whether there are patients who were treated elsewhere for sequelae of TPF surgery. However, the use of real-world data can also be considered a strength of the study, as it reflects routine clinical practice. Although confounding factors were adjusted in the analysis, the influence of unmeasured or unknown variables cannot fully be excluded. Detailed information regarding specific fixation characteristics, including locking versus non-locking constructs and the surgical approaches used, could not be reliably assessed retrospectively. Although available surgical characteristics were included in the analysis, residual heterogeneity in fixation strategy may have influenced the risk of LoR. Furthermore, DEXA-derived T-scores were only available for a small subset of patients, limiting the assessment of bone mineral density as a potential factor associated with LoR. Although age was independently associated with LoR, the influence of age-related changes in bone quality could not be evaluated. The duration of follow-up varied between patients, with some lacking long-term imaging beyond the initial phase of fracture healing. This may have led to an underestimation of LoR in the long term. Lastly, clinical and functional outcomes were not routinely collected, preventing correlation of radiographic findings with functional results.

## Conclusion

In this retrospective study of 213 patients, several factors were associated with loss of reduction. Using strict radiological criteria, patient age, posterior column involvement, greater preoperative step-off, and residual step-off and condylar widening were independently associated with increased odds of LoR, after adjustment for confounding. These findings underscore the importance of achieving optimal fracture reduction and fixation to minimize the risk of LoR, especially in older patients and complex posterior fracture patterns.

## Data Availability

No datasets were generated or analysed during the current study.
